# Metaproteomic Analysis of an Oral Squamous Cell Carcinoma Dataset Suggests Diagnostic Potential of the Mycobiome

**DOI:** 10.3390/ijms24021050

**Published:** 2023-01-05

**Authors:** Steven He, Rajdeep Chakraborty, Shoba Ranganathan

**Affiliations:** Applied Biosciences, Faculty of Science and Engineering, Macquarie University, Sydney 2109, Australia

**Keywords:** microbiome, oral cancer, metaproteomics, mycobiome

## Abstract

Oral squamous cell carcinoma (OSCC) is the most common head and neck malignancy, with an estimated 5-year survival rate of only 40–50%, largely due to late detection and diagnosis. Emerging evidence suggests that the human microbiome may be implicated in OSCC, with oral microbiome studies putatively identifying relevant bacterial species. As the impact of other microbial organisms, such as fungi and viruses, has largely been neglected, a bioinformatic approach utilizing the Trans-Proteomic Pipeline (TPP) and the R statistical programming language was implemented here to investigate not only bacteria, but also viruses and fungi in the context of a publicly available, OSCC, mass spectrometry (MS) dataset. Overall viral, bacterial, and fungal composition was inferred in control and OSCC patient tissue from protein data, with a range of proteins observed to be differentially enriched between healthy and OSCC conditions, of which the fungal protein profile presented as the best potential discriminator of OSCC within the analysed dataset. While the current project sheds new light on the fungal and viral spheres of the oral microbiome in cancer in silico, further research will be required to validate these findings in an experimental setting.

## 1. Introduction

Oral cancer carries a heavy global burden, being the most common head and neck malignancy worldwide, with an observed 377,713 new cases in 2020, and one of the leading causes of death in India among males [1,2]. Oral squamous cell carcinoma (OSCC) is the predominant form of oral cancer, with many aetiological factors, including age, alcohol, tobacco use, and the traditional chewing of areca nuts in regions such as South Central Asia [3,4,5].

Among OSCC patients, the overall 5-year survival rate is an estimated 40–50% [6]. However, intervention and treatment in patients presenting with early stage oral cancer show drastically improved outcomes, with an estimated 78–92% 3-year survival rate [7]. As with all cancers, proliferation and apoptotic pathways play a key role in OSCC [8], with recent evidence demonstrating the ability of oral bacterial microfilms to modulate cell proliferation [9]. This, coupled with a growing body of evidence, suggests that the oral microbiome may be implicated in the development of OSCC [10]. Thus, these oral microorganisms may exhibit potential as biomarkers to support OSCC disease diagnosis.

The association between microorganisms and carcinogenicity was first demonstrated in the 1990s through Helicobacter pylori [11], with modern estimates now suggesting H. pylori is causally related to 60–90% of all gastric cancers [12]. Microbiome research has suggested three likely primary mechanisms for the promotion of microbial carcinogenesis, including inflammation and activation of Toll-like receptors; secretion of microbial genotoxins, such as colibactin; and alteration of metabolic states [13]. With the advent of next-generation sequencing, many researchers are now able to probe the associations between cancer and the microbiome using culture-free metagenomic approaches, including, in particular, 16S rDNA sequencing. For OSCC, many different species, including periodontitis-related pathogens, from such experimental studies reportedly correlate with OSCC presence, with the most consistently observed being Fusobacterium nucleatum [10,14,15,16]. In a murine model of 4-nitroquinoline-1-oxide-induced oral carcinogenesis, mice that were repeatedly infected with Fusobacterium nucleatum and the additional periodontal pathogen Poryphyromonas gingivalis were observed to have larger and more invasive tumours, accompanied by increased expression of IL-6, phospho-STAT3, and cyclin D1, which have roles in inflammation and cellular proliferation [17].

In comparison to bacteria, the fungal and viral components of the microbiome in OSCC have been largely understudied and remain relatively poorly characterised. For viruses, much of the literature has focused on human papillomavirus (HPV), which has been reported as a classical risk factor for OSCC [18], due in part to the histological similarity between the oral and vaginal mucosa [19], the high prevalence of HPV in OSCC [20], and the ability of HPV to immortalise human keratinocytes in vivo [21]. While strong evidence links HPV to oropharyngeal cancer [22], its role in OSCC remains controversial, as many discrepancies exist within the literature; HPV DNA presence in potentially oral malignant lesions has been reported to range from 0 to 85% [20], with additional reports of HPV-positive cancer rates as low as 13% [23]. It has been alternatively postulated that HPV infection may be opportunistic and not necessarily a cause of OSCC carcinogenesis [24]. HPV-negative OSCC tumours remain far more prevalent, accounting for approximately 75% of all head and neck squamous cell carcinoma (HNSCC) cases, and generally result in worse prognosis than HPV-positive tumours [25]. Epstein–Barr virus (EBV) is an additional focus point in OSCC viral research, albeit to a lesser degree, as the oral cavity is the primary site for its transmission and persistence [26]. Despite >90% of adults being infected [27], EBV-associated oral cancers are relatively rare, with some evidence suggesting infection may promote tumour progression and a metastatic phenotype [26]. Regarding fungi, some of the most compelling evidence implicating carcinogenesis comes from the *Candida* species, which have been observed to be more prevalent in the saliva of OSCC patients compared to healthy controls [28]. *Candida albicans* cultured from patients having potentially malignant oral mucosal disorders has been demonstrated to produce carcinogenic levels of acetaldehyde (>100 μM) by gas chromatography, with this effect exacerbated in isolates taken from patients who reported tobacco and alcohol use, both of which are risk factors for OSCC [29]. In fact, one metagenomic study evaluated mycobiome composition in OSSC and observed enrichment of the genus *Candida*, as well as *Hannaella* and *Gibberella* [30].

The current study analysed a publicly available, HPV-negative, MS dataset of head and neck squamous cell carcinoma (HNSCC; including OSCC), reported by Huang et al. [31]. We implemented a metaproteomic approach using the Trans-Proteomic Pipeline (TPP) [32]. As a large number of oral cancer studies have employed metagenomic approaches, which only provide information of functional potential [10,14,15,33,34], the use of metaproteomics identifies expressed proteins and acts as an orthogonal technique for further validation of the existing literature. Given the disproportionate focus on bacteria, the current study also analysed viral and fungal proteins in the OSCC MS dataset, with the aim of identifying microbial proteins and species with potential as diagnostic OSCC biomarkers for OSCC.

## 2. Results

A total of 5742, 3459, and 5594 significant viral, bacterial, and fungal peptides were identified, respectively (Supplementary File S1). At the protein level, 96, 201, and 934 viral, bacterial, and fungal proteins, respectively, were identified (Supplementary File S2). From the protein data, species inference was carried out as a first step, followed by statistical testing for differentially abundant proteins, and then heatmapping.

### 2.1. An Increased Overall Microbial Species Diversity Is Observed in OSCC Conditions

**Figure 1:**
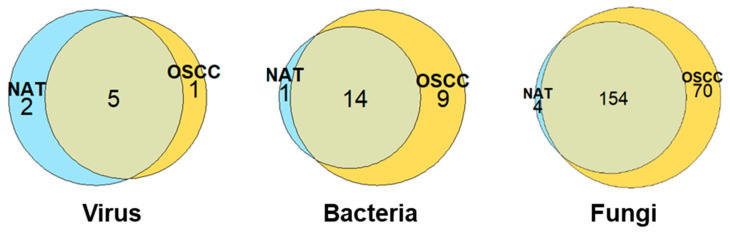
Distribution of microbial species between NAT and OSCC conditions: Venn diagrams displaying number of viral, bacterial, and fungal species identified by at least two unique proteins and their allocation to NAT, OSCC, or both conditions based on log-fold change cut-offs. Species inferred by proteins with a mean intensity log_2_ fold-change of ≤−1 or ≥1 were assigned to NAT and OSCC conditions, respectively, with those between this range being considered present in both conditions.

The greatest microbial diversity was observed among fungal species, with a total 228, 24, and 8 species identified for fungi, bacteria, and viruses, respectively. The distributions of these species between the NAT and OSCC conditions can be viewed in Figure 1.

**Figure 2:**
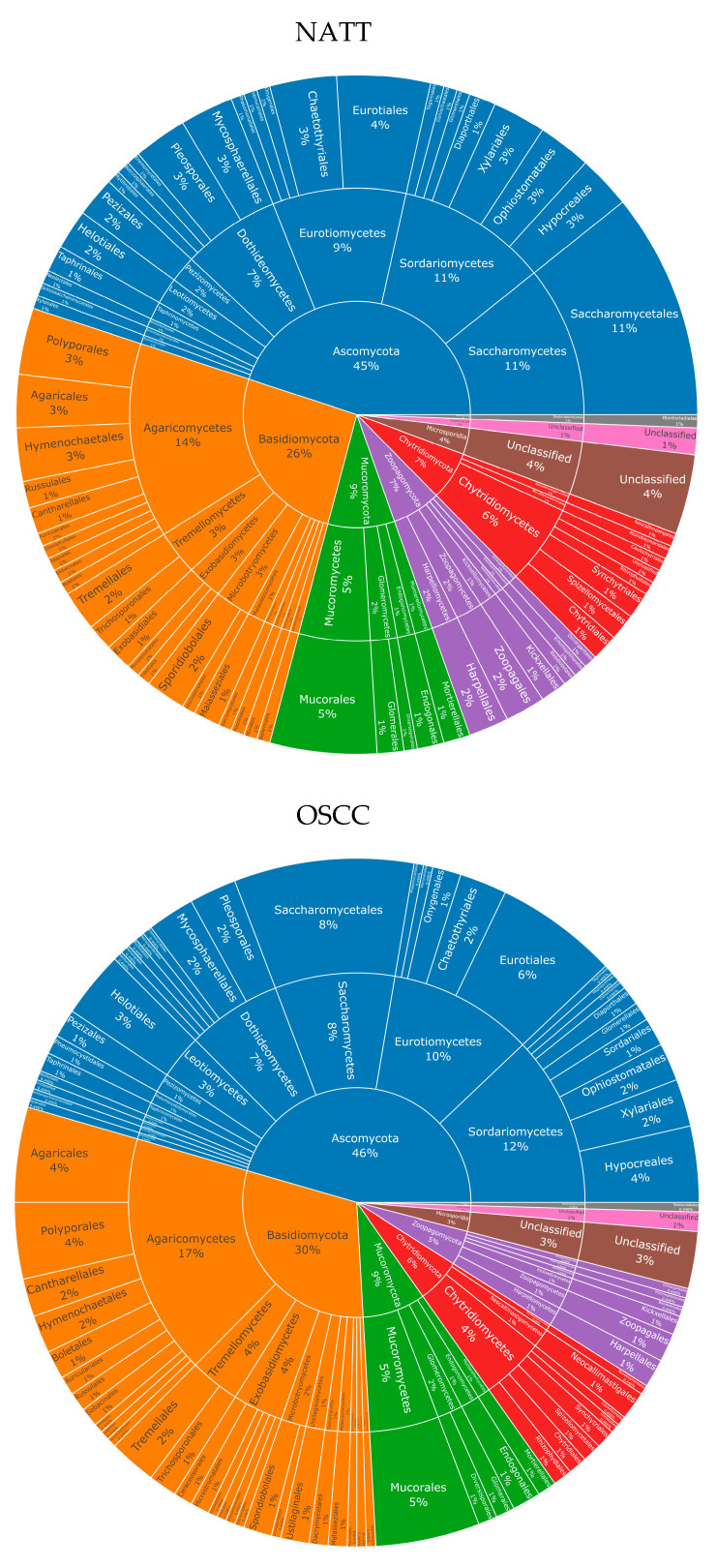
Fungal taxonomy in NAT and OSCC patient samples: Sunburst plots showing inferred fungal composition in NAT (top) and OSCC (bottom) patient samples. Classification level from inner to outermost radials: Phylum, Class, Order. Colours represent species’ phylum, while percentage values represent proportion within a given classification level. Taxonomic sunburst plots for bacterial and viral species can be viewed in Supplementary Figures S1 and S2. List of all species can be viewed in Supplementary File S2.

For fungi, eight phyla were observed in both NAT and OSCC conditions: Ascomycota, Basidiomycota, Mucoromycota, Chytridiomycota, Zoopagomycota, Microsporidia, Cryptomycota, and Blastocladiomycota. The percentage composition for these were 45, 26, 9, 7, 7, 4, 1, and 1, respectively, for NAT, and 46, 30, 9, 6, 5, 3, 1, and >1, respectively, for OSCC (Figure 2).

For bacteria, four main phyla were identified in both NAT and OSCC conditions; Firmicutes, Proteobacteria, Actinobacteria, and Bacteroidetes, with a fifth phylum, Fusobacteria, only being observed in the OSCC profile. The percentage composition for these were 47, 20, 27, 7, and 0, respectively, for NAT, and 39, 26, 13, 13, and 9, respectively, for OSCC. (Supplementary Figure S1).

Only two main kingdoms were identified in both NAT and OSCC conditions for viruses: *Bamfordvirae* and *Heunggongvirae*, as well as an “Unclassified” category. The percentage composition for these were 43, 29, and 29, respectively, for NAT, and 17, 33, and 50, respectively, for OSCC (Supplementary Figure S2).

### 2.2. Identification of Differentially Abundant Fungal Proteins

**Figure 3:**
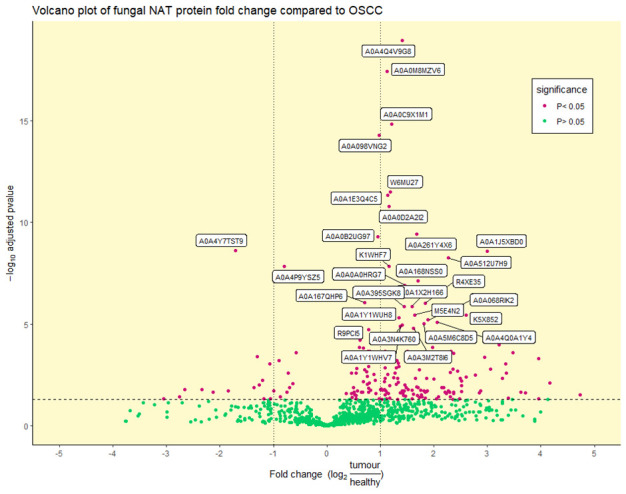
Volcano plot of differentially abundant fungal proteins: Differentially (purple) and non-differentially (green) abundant fungal proteins between OSCC and NAT patient samples (adjusted *p*-value < 0.05) determined by statistical *t*-testing using Benjamini–Hochberg correction. The log_2_ fold change reflects the ratio of normalised TMT-label intensities between tumour OSCC and healthy NAT conditions. The horizontal dotted line represents an adjusted *p*-value of 0.05. Vertical dotted lines represent ± 1 log_2_ fold change. UniProt accession numbers are shown for the top 30 most significant proteins.

Benjamini–Hochberg statistical *t*-testing identified a total of 6 out of 97, 14 out of 201, and 196 out of 937 significantly differentially abundant viral, bacterial, and fungal proteins, respectively (adjusted *p*-value < 0.05) (Figure 3 and Supplementary Figure S3). Due to the low number of significant viral and bacterial proteins, reflecting the low viral and bacterial diversity seen in Figure 1, we will focus on the fungal microbiome. Of the top 30 most significantly differentially abundant proteins, 2 were enriched in the NAT condition, with the remaining 28 enriched in the OSCC condition (Figure 3 and Table 1).

### 2.3. Hierarchical Clustering Reveals Diagnostic Potential of Fungal Microbiome

**Figure 4:**
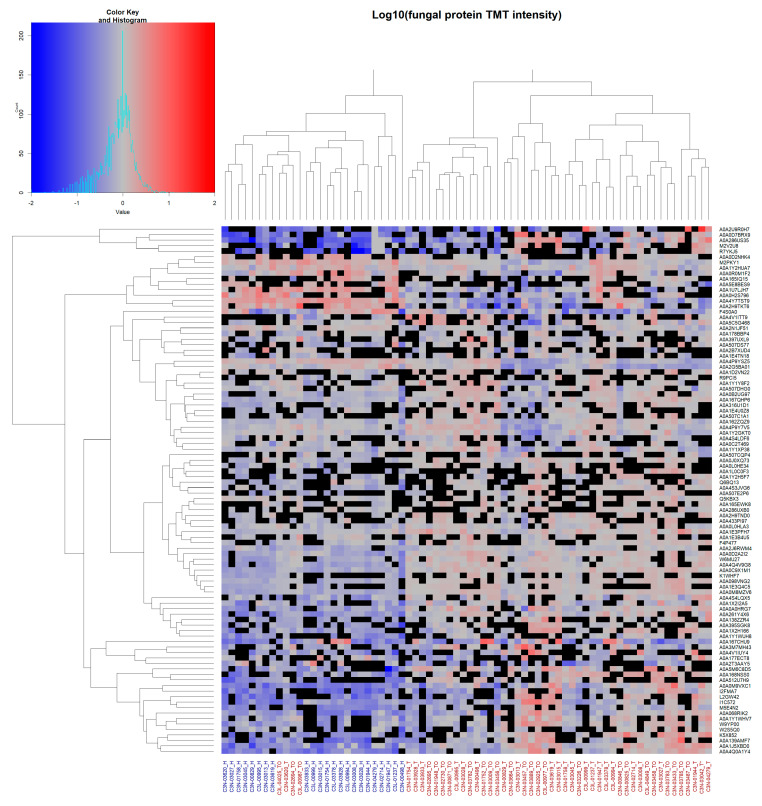
Unsupervised hierarchical clustering of fungal protein data: Unsupervised clustering of fungal proteins observed in ≥24 total patient samples. Complete linkage clustering was used with Euclidean distance measures. Cells show log10-normalised TMT-label intensity with blue and red indicating reduced and increased intensity, respectively, relative to the experimental reference sample. Black cells indicate missing values. Column labels represent either healthy NAT (blue labels) or tumour OSCC (red labels) patient samples. Protein UniProt accession numbers are shown for each row.

Unsupervised hierarchical clustering of all identified fungal proteins observed in ≥24 of patient samples (~≥50%; total of 96 fungal proteins) was performed (Figure 4).

Here, a separation between NAT and OSSC samples was observed, with only four OSCC samples clustering together with the healthy NAT patient samples. Clustering for viral and bacterial proteins was similarly performed, although no clear separation was observed due to the low number of proteins (Supplementary Figure S4).

## 3. Discussion

### 3.1. Enrichment of Nucleocytoplasmic Large DNA Viruses in the Oral Virome

Regarding viruses, statistical testing identified only six viral proteins, which significantly differed in abundance between NAT and OSCC samples, with half of these being uncharacterised proteins and the other half corresponding to proposed replication and heat shock proteins. Four of the six proteins mapped back to members of the *Phycodnaviridae* family, a group of giant viruses referred to as nucleocytoplasmic large DNA viruses (NCLDVs) [35]. Microalgae serve as the natural hosts for NCLDVs, though many foods for human consumption use microalgae to fortify protein content and supplement nutrition [36]. The enrichment of these proteins in the OSCC condition suggest another possible association between OSCC and diet, as microalgae food safety, especially in the context of contaminants, is not well characterised [37]. However, this would require further investigation, given the low overall number of viral proteins observed here. Of note, no HPV proteins could be detected at all, remaining consistent with the screening by Huang et al. to ensure only HPV-negative samples were used in the analysed dataset.

### 3.2. Periodontal Pathogens and Opportunistic Bacteria Are Enriched in OSCC

Statistical testing of bacterial protein fold-changes revealed 14 proteins that were predominantly significantly enriched in OSCC samples. Twelve of these mapped back to individual species, with only one species being inferred by two proteins here. The majority of these are normal constituents of the human microbiome and have low virulence, but act as opportunistic pathogens in immunocompromised individuals. This includes *Paracoccus yeei* [38], *Pseudomonas luteola* [39], *Staphylococcus epidermidis* [40], *Cardiobacterium hominis* [41], and *Staphylococcus lugdunensis* [42]. These bacteria likely represent “passengers” in the “passenger-turning-driver” model [43]. Most notably, *S. lugduenensis* represents a normal human commensal, which flourishes during oral infection, expressing hemolysin virulence factors that promote further inflammatory states that may favour cancer growth [44,45,46]. In addition to these opportunistic pathogens, other inferred species include *Variovorax paradoxus*, *Eubacterium minutum*, and *Microbacterium flavescens*, which are notably enriched in periodontitis [47,48,49,50]. *V. paradoxus*, in particular, was the only species here inferred by two proteins with documented biofilm-formation phenotypes [51]. There is reasonable evidence suggesting periodontitis increases the risk of developing oral cancer [52], with one meta-analysis identifying a 2–5 fold increased oral cancer risk associated with periodontitis [53].

### 3.3. An Unexpected Fungal Diversity Is Observed in the OSCC Patient Samples

Current research on fungi within the oral microbiome has been largely eclipsed by its bacterial members, though advancements in omics approaches has renewed interest in this area, with high-throughput sequencing revealing a complex fungal microbiome, or mycobiome [54].

Existing literature suggests the oral mycobiome is predominated by fungi from the phylum Ascomycota, followed by Basidiomycota [30], and this trend was observed in the current study, where these two phyla predominated in the oral mycobiome composition in both NAT and OSCC tissue. However, the current study additionally identified substantial diversity from the additional phyla *Mucoromycota*, *Zoopagomycota*, *Chytridioomycota*, and *Microsporidia*, with the overall proportion of these phyla remaining relatively stable between NAT and OSCC.

Unexpectedly, the current results identified a greater number of fungal proteins and species, compared to both bacteria and viruses. Though fungi have been described as comprising <0.1% of the human microbiome, this estimate is based on cfu [55] and likely under-estimates the true fungal frequency. These cfu measurements require microbial cultures, and a large proportion of fungi from the human microbiome are unable to be cultured [56,57,58]. In addition, despite being numerically underrepresented, the generally larger cell size of fungal species has been speculated to contribute a proportionally larger amount of biomass [55]. It is also possible that the increased number of identified fungal proteins was due to the use of surgical antiseptics, such as chlorhexidine, povidone iodine, and, less commonly, alcoholic disinfectants [59,60,61]. Alcohols remain effective against bacteria, but demonstrate low efficacy against fungi and fungal spores [60,62]. Conversely, povidone iodine and chlorhexidine exhibit greater fungicidal activity, with povidone iodine demonstrating additional efficacy against spores [59,60,63,64]. Due to their prevalence, many experiments have demonstrated the efficacy of povidone iodine and chlorhexidine against *Candida* species specifically [64,65,66,67], likely explaining the lack of *Candida* species identified in the current study. Limited information, however, could be found regarding the efficacy of these antiseptics on other fungal genera, with the additional possibility that surface cleaning of the biopsy site is ineffective against intraepithelial fungal species that have penetrated the mucosal layer [68].

### 3.4. Fungal Proteins Implicate Pathogens Capable of Soft Tissue Damage

Following statistical testing, 196 fungal proteins were identified as being significantly differentially abundant between the NAT and OSCC conditions. While a large number of fungal species were inferred, many of these were poorly characterised, with no documented pathogenicity or documented interactions in the human oral cavity. Similar to identified bacteria, several inferred fungi were documented to be, or be associated with, opportunistic pathogens, including *Verruconis gallopava* [69], *Syncephalastrum racemosum* [70], and *Dimargaris cristalligena* [71,72], all of which were inferred to be more abundant in OSCC tissue. Some more notable and well-characterised fungal species inferred from proteins include *Lichteimia corymbifera*, *Malassezia sympodialis*, and *Paracoccidioides brasiliensis*. *L. corymbifera* is capable of causing mucormycosis fungal infection, which can lead to ulceration of the oral cavity [73,74], and was recently examined to be strongly associated with mobile tongue OSCC in a recent metagenomic study examining tumour tissue against non-tumor tissue controls [75]. *Lichteimia* species are one of the predominant causative agents of mucormycosis in Europe, with maxillo-facial and pulmonary infections as common clinical presentations [76,77]. Clinical patient studies have additionally observed mucormycosis to be more commonly associated with hematological malignancies, such as acute leukemia and lymphoma, though this is possibly a result of opportunistic infection rather than tumour initiation [77,78]. *M. sympodialis* is considered to be a normal commensal of the human oral microbiome [79], though a recent in vivo study utilising mouse tumour models demonstrated increased *Malassezia* abundance in pancreatic tumour mice compared to controls, with further knockout mice suggesting that tumour progression is driven by activation of the mannose-binding lectin—C3 complement cascade [80]. *P. brasiliensis* is a fungal yeast capable of paracoccidioidomycosism fungal infection, which may present as oral lesions and other soft-tissue damage [81,82]. In patients with both paracoccidioidomycosism and OSCC, these malignancies often occur in the same region or adjacent tissues, highlighting a potential role of this fungus in cancer aetiology [83]. It has been hypothesised that continuous stimulation of epithelial cells may predispose these cells to malignant transformation, and that these may persist due to fungal-impaired macrophage and natural killer cell activity [84]. This association, however, is not conclusive due to the overall low number of experimental studies examining this effect [84]. In a retrospective study examining patients diagnosed with both paracoccidioidomycosism and cancer, 62.5% presented lung tumours, with the majority of these being classified as squamous cell carcinoma [85]. While the authors conclude that paracoccidioidomycosism appears to increase the risk of cancer, particularly lung cancer, there is little research on whether paracoccidioidomycosism infection has a role in tumor initiation, or if it acts as a “passenger” following oncogenesis.

It is surprising to note that, among the inferred species enriched in OSCC, Candida albicans, a species that has been reported to dominate the OSCC mycobiome landscape [30], was not observed here. This is, again, likely due to antiseptic use during specimen collection, as *C. albicans* is observed to be susceptible to povidone iodine, chlorhexidine, and alcohol disinfectants, such as isopropanol [64,65,66,67,86].

### 3.5. Clustering of the Mycobiome Protein Profile Shows Diagnostic Potential

When compared to bacteria and viruses, unsupervised hierarchical clustering of fungal proteins identified in at least 50% of patients provided the greatest discrimination between NAT and OSCC samples. Apart from four OSCC samples, all NAT samples clustered together based on this fungal protein profile. This superior discrimination is likely achieved through the high identification rate of fungal proteins, potentially attributed to the use of chemical disinfectants that have biased the overall microbial composition. Nonetheless, it is still promising to identify that the oral mycobiome, which has been understudied, may be used as potential indicators of oral carcinogenesis.

Further research into the fungal composition of the oral cavity is needed, with investigations using unbiased samples being potentially capable of identifying novel biomarkers for the diagnosis of OSCC.

## 4. Methods

### 4.1. Data Collection

The selected HPV-negative MS dataset of head and neck squamous cell carcinoma (HNSCC; including OSCC) was downloaded from the Clinical Proteomic Tumour Analysis Consortium public repository (accessed August 2021), as reported by Huang et al. [31]. This portal, however, has since been retired, with the data now accessible in the Proteomic Data Commons repository with the identifier PDC000221 (https://pdc.cancer.gov/pdc/study/PDC000221; accessed on 20 January 2022). Data-dependent acquisition (DDA) was used for the generation of this data.

Huang et al. [31] collected 109 treatment-naive primary tumours and matched blood samples from tumours of samples from mainly the oral cavity and the larynx, with few samples from the lip, hypopharynx, and otopharnyx. Sixty-six tumours had matched normal adjacent tissues (NATs). Specimen inclusion was based on the maximal percent in the pathology criteria and best weight, with clinical details of all samples provided in Huang et al. [31]: Table S1. One sample was excluded as it was HPV-positive. Information regarding erosive/productive phenotypes was not available. These samples were tandem mass tag (TMT)-labelled (11-plex) with a reference standard included in the first channel of each TMT set, produced by pooling prepared peptide solutions from 87 HNSCC and 50 healthy, normal, adjacent tissue (NAT) samples [31].

We have included only HPV-negative samples from the oral cavity for the current analysis, comprising 49 total patient samples, of which 23 had matched normal adjacent tissue (NAT) and 26 had tumour tissue only. While tumour stage information was available, analysis was performed on all cancer stages together, compared to non-cancer conditions, due to large differences in sample numbers between the tumour stage groups (Stage I: 4, Stage II: 12, Stage III: 10, and Stage IV: 23).

Viral and fungal reference proteomes were downloaded from UniProt (https://www.uniprot.org/proteomes; release number 2021_03, accessed on 2 June 2021) in FASTA format. Bacterial reference sequences were downloaded from the expanded Human Oral Microbiome Database (eHOMD) (version 9.1.4; updated 9 September 2020) [87,88]. Details of each can be seen in Table 2.

For fungal sequences, due to the large search space, CD-HIT software was implemented to perform clustering at 90% sequence identity [89]. An approximate 1.32-fold reduction in search space was achieved, with 8,370,376 initial fungal protein entries reduced to 6,326,765 protein clusters. An additional human reference proteome was downloaded from UniProt (Release 2021_04, 29 September 2021) and appended to each microbial database to improve false discovery rate (FDR) performance.

### 4.2. Trans-Proteomic Pipeline Analysis

A modified version of our recently developed generic protocol was implemented [90]. TPP (ver. 6.0.0) was used for primary analysis of the publicly available MS data (http://tools.proteomecenter.org/TPP.php; accessed on 20 January 2022) [91]. TPP is a freely available platform for the complete analysis of MS data, including software for file conversion, database searching, peptide validation, and protein inference. Proprietary raw files were converted in TPP’s MSConvert to mzML before database searching in TPP’s Comet. Preset mass modifications of 229.162932 to both the peptide N-termini and lysine residues with additional clearing of the 125.5–131.5 *m*/*z* range were implemented to account for TMT-labelling. Defaults were also used for remaining Comet parameters, with an optimised peptide mass tolerance of 5 ppm implemented. The corresponding pepXML output files were subsequently analysed in TPP’s PeptideProphet for peptide validation. Accurate mass binning implemented using ppm, a minimum peptide length of 9 amino acids, and known decoy hits were used to pin down the negative distribution (as detailed in [90]). TPP’s Libra software was also utilised using the default values for TMT-11 channel labelling, with intensity values normalised against the reference standard. The PeptideProphet outputs were then analysed in ProteinProphet for protein inference. Each TMT reaction (comprising 24 fractions) searched against a particular database was analysed separately, using default settings and a similar implementation of Libra software for TMT 11-channel labelling.

### 4.3. Secondary Analysis and Visualisation

Data exported from ProteinProphet were processed using R (version 4.1.2) in RStudio (version 2021.09.1, build 372) software. Protein assignments with low ProteinProphet probability scores were filtered to maintain a 1% protein level FDR, followed by filtering of human proteins, and then proteins that could not be uniquely identified to a single species. In addition, only proteins that were inferred by at least 2 unique peptides (minimum length of 9 amino acids) and observed in ≥2 matched patient samples were retained.

Filtered high-probability protein assignments were used as best indicators of species inference. Assignment to OSCC, NAT, or both conditions was based on log_2_ fold-change cut-offs (*log*_2_$\left(\frac{OSCC\;intensity}{NAT\;intensity}\right)$). Species inferred by proteins with an intensity log_2_ fold-change ≤−1 were assigned to the NAT condition, whilst conversely, species inferred by proteins with intensity log_2_ fold-change ≥1 were assigned to the OSCC condition. Species inferred by proteins between these cut-offs were allocated to a “core” condition present in both OSCC and NAT samples. For species inferred by more than one protein, the mean log_2_ fold-change of all proteins was used to assign the species to a particular condition. Inferred species were matched against the NCBI taxonomy database (https://ftp.ncbi.nlm.nih.gov/pub/taxonomy/new_taxdump; accessed on 1 February 2022), and taxonomic information was visualised as sunburst plots using the ‘plotly’ package [92].

The ‘gplots’ package was used to perform unsupervised hierarchical clustering of proteins that were observed in ≥50% of patient samples [93]. Complete linkage clustering was used with Euclidean distance, with an additional log_10_ transformation of the protein fold-change data, and these data were visualised as heatmaps.

Statistical pairwise *t*-testing was performed on all significantly identified proteins to determine differentially abundant proteins between NAT and OSCC based on the fold-change of Libra values (corresponding to the relative intensity of the TMT-labels). Adjusted *p*-values were calculated using Benjamini–Hochberg correction, with an adjusted *p*-value cut-off of *p* < 0.05 for significance. Results of this statistical analysis were visualised as volcano plots using the ‘ggplot2′ package [94].

## 5. Conclusions

The current study examined the oral microbiome of a public oral cancer dataset using a metaproteomic bioinformatic approach. To the best our knowledge, this is the first such report with low numbers of viral and, surprisingly, bacterial species identified, complemented by a high fungal diversity. This was likely due to biases within the dataset, including the use of surface disinfectants. Though poorly characterised, fungal proteins differentially abundant in OSCC implicated several species capable of causing ulceration and soft tissue damage, including *V. gallopava*, *S. racemosum*, and *D. cristalligena*. Hierarchical clustering of the fungal protein profile also resulted in the best separation between the NAT and OSCC conditions, suggesting that the understudied oral mycobiome may have diagnostic biomarker potential for OSCC. Future experiments withholding the use of surface disinfectants would be needed to further validate these findings. A multi-omics approach combining metagenomic, metatranscriptomic, and metaproteomic experiments on the same set of patient samples—balancing all cancer stages and, specifically, from erosive and productive phenotypes—would provide biomarkers with increased confidence. Additionally, potential further work comparing the oral cancer microbiome to other epithelial cancers would allow for the identification of common microbial species between these that may also provide insight into disease aetiology.

## Figures and Tables

**Table 1:** Significantly differentially abundant fungal protein details: Information on the top 30 significant proteins from Figure 3. Information for all significant proteins can be viewed in Supplementary File S3. UniProt accession numbers, protein description, corresponding species, log_2_ fold change, and adjusted *p*-values are shown. Entries are sorted by ascending adjusted *p*-value.

Accession	Protein Description	Fungal Species	Log2 Fold Change	Adjusted *p*-Value
A0A4Q4V9G8	HET domain-containing protein	*Monosporascus* sp. *MG133*	1.41	1.17 × 10^−19^
A0A0M8MZV6	Tubulin beta chain	*Escovopsis weberi*	1.13	3.94 × 10^−18^
A0A0C9X1M1	Unplaced genomic scaffold K443scaffold_229, whole genome shotgun sequence	*Laccaria amethystina*	1.22	1.49 × 10^−15^
A0A098VNG2	Tubulin beta chain	*Mitosporidium daphniae*	0.98	5.31 × 10^−15^
W6MU27	ERF-3	*Kuraishia capsulata*	1.19	3.16 × 10^−12^
A0A1E3Q4C5	Tubulin beta chain	*Lipomyces starkeyi*	1.14	4.80 × 10^−12^
A0A0D2A2I2	Endoplasmic reticulum chaperone BiP	*Verruconis gallopava*	1.16	1.66 × 10^−11^
A0A261Y4X6	Adenine phosphoribosyltransferase	*Bifiguratus adelaidae*	1.68	3.66 × 10^−10^
A0A0B2UG97	Tubulin beta chain	*Ordospora colligata*	0.95	5.10 × 10^−10^
A0A4Y7TST9	Actin-1	*Coprinellus micaceus*	−1.70	2.45 × 10^−09^
A0A1J5XBD0	Actin	*Amphiamblys* sp. *WSBS2006*	3.00	2.57 × 10^−09^
A0A512U7H9	Uncharacterized protein	*Metschnikowia* sp. *JCM 33374*	2.27	5.62 × 10^−09^
K1WHF7	DNA-directed RNA polymerase subunit	*Marssonina brunnea f.* sp. *multigermtubi*	1.17	1.42 × 10^−08^
A0A4P9YSZ5	Glyceraldehyde-3-phosphate dehydrogenase	*Syncephalis pseudoplumigaleata*	−0.80	1.42 × 10^−08^
A0A168NSS0	SAM_MT_RSMB_NOP domain-containing protein	*Absidia glauca*	1.71	7.38 × 10^−08^
A0A0A0HRG7	Actin	*Paracoccidioides brasiliensis*	1.46	1.25 × 10^−07^
A0A167QHP6	Uncharacterized protein	*Phycomyces blakesleeanus*	0.70	8.77 × 10^−07^
R4XE35	DNA-directed RNA polymerase subunit	*Taphrina deformans*	1.84	9.27 × 10^−07^
A0A1X2H166	Eukaryotic peptide chain release factor subunit 1	*Syncephalastrum racemosum*	1.60	1.32 × 10^−06^
A0A395SGK8	Non-ribosomal peptide synthetase	*Fusarium longipes*	1.44	1.32 × 10^−06^
K5X852	WD_REPEATS_REGION domain-containing protein	*Phanerochaete carnosa*	2.60	3.48 × 10^−06^
M5E4N2	Tryptophanyl-tRNA synthetase	*Malassezia sympodialis*	1.64	3.48 × 10^−06^
A0A1Y1WUH8	Beta-glucosidase	*Anaeromyces robustus*	1.34	4.96 × 10^−06^
A0A068RIK2	Phosphoglycerate kinase	*Lichtheimia corymbifera*	1.89	6.27 × 10^−06^
A0A4Q0A1Y4	14-3-3 domain-containing protein	*Dimargaris cristalligena*	2.06	7.94 × 10^−06^
A0A5M6C8D5	ADP-ribosylation factor	*Kwoniella shandongensis*	1.82	9.64 × 10^−06^
A0A3N4K760	Tubulin alpha chain	*Morchella conica*	1.41	1.10 × 10^−05^
A0A1Y1WHV7	Dynein heavy chain, cytoplasmic	*Linderina pennispora*	1.37	1.27 × 10^−05^
A0A3M2T8I6	Uncharacterized protein	*Aspergillus* sp. *HF37*	1.62	1.63 × 10^−05^
R9PCI5	Tubulin beta chain	*Pseudozyma hubeiensis*	0.78	1.90 × 10^−05^

**Table 2:** Microbial reference database details: Information regarding reference database sources, including number of entries, size, URL, and version information.

Organism Type	No. of Reference Proteomes	No. of Protein Entries	Size (GB)	URL/Directory
Virus	10,062	517,610	0.18	https://ftp.uniprot.org/pub/databases/uniprot/current_release/knowledgebase/reference_proteomes/Viruses/ (release no. 2021_3; accessed on 2 June 2021)
Fungi	784	8,370,376	4.61	https://www.uniprot.org/uniprot/?query=proteome%3a(taxonomy%3a%22Fungi+%5b4751%5d%22+AND+reference%3ayes) (release no. 2021_3; accessed on 2 June 2021)
Bacteria	2087	5,044,213	1.89	http://homd.org/ftp/genomes/PROKKA/current/faa/ (ver 9.1.4: accessed on 9 September 2020)

## Data Availability

The R code used for the secondary analysis of the MS data can be found at GitHub through the following link: https://github.com/hehestevenhe/MDPI_OSCC_microbiome/tree/main/RFiles (accessed on 2 June 2021).

## Supplementary Materials

The following supporting information can be downloaded at: https://github.com/hehestevenhe/MDPI_OSCC_microbiome/tree/main/SupplementaryFiles.
